# Dual B- and T-cell de-immunization of recombinant immunotoxin targeting mesothelin with high cytotoxic activity

**DOI:** 10.18632/oncotarget.9171

**Published:** 2016-05-04

**Authors:** Ronit Mazor, Masanori Onda, Dong Park, Selamawit Addissie, Laiman Xiang, Jingli Zhang, Raffit Hassan, Ira Pastan

**Affiliations:** ^1^ Laboratory of Molecular Biology, Center for Cancer Research, National Cancer Institute, National Institutes of Health, Bethesda, Maryland, USA; ^2^ Thoracic and GI Oncology Branch, National Cancer Institute, National Institutes of Health, Bethesda, Maryland, USA; ^3^ New Business Development Department, Medytox Inc., Bundang-gu, Seongnam-si, Gyeonggi-do, South Korea

**Keywords:** epitope, immunogenicity, rational design, mesothelioma, pancreatic cancer

## Abstract

Recombinant immunotoxins (RITs) are genetically engineered proteins being developed to treat cancer. They are composed of an Fv that targets a cancer antigen and a portion of a protein toxin. Their clinical success is limited by their immunogenicity. Our goal is to produce a new RIT that targets mesothelin and is non-immunogenic by combining mutations that decrease B- and T-cell epitopes. Starting with an immunotoxin that has B-cell epitopes suppressed, we added mutations step-wise that suppress T-cell epitopes. The final protein (LMB-T14) has greatly reduced antigenicity as assessed by binding to human anti-sera and a greatly decreased ability to activate helper T-cells evaluated in a T-cell activation assay. It is very cytotoxic to mesothelioma cells from patients, and to cancer cell lines. LMB-T14 produces complete remissions of a mesothelin expressing cancer (A431/H9) xenograft. The approach used here can be used to de-immunize other therapeutic foreign proteins.

## INTRODUCTION

Recombinant immunotoxins (RITs) are antibody-toxin fusion proteins developed for cancer therapy. SS1P is a RIT composed of a Fv that targets mesothelin and a 38-kDa fragment of *Pseudomonas* exotoxin A (PE38).SS1P was developed to treat a variety of mesothelin expressing tumors; these include mesothelioma, ovarian, pancreatic, lung, stomach and cervical cancer [1–4]. In a phase 1 clinical trial in which SS1P was given QODx3 every 21 days, neutralizing antibodies formed after the first cycle in 90% of patients and no major clinical responses were observed [5]. However, when SS1P was used in combination with an immunosuppressive regimen of cytoxan and pentostatin to kill B- and T-cells, additional treatment cycles could be given and major tumor responses were observed in several patients with advanced refractory mesothelioma [6]. This indicates that producing less immunogenic RITs should allow more treatment cycles and more clinical responses.

Formation of anti-drug antibodies is a major problem in the development of protein therapeutics [7] and specifically foreign proteins like a bacterial toxin [8]. The antibodies involved in the immunogenicity response against SS1P mostly react with PE38, the toxin portion of the RIT [8]. The formation of high affinity IgG is primarily dependent on activation of three cellular entities: Antigen presenting cells that process the antigen and present it to T-cells, T-helper cells that secrete cytokines that are required for class switching and affinity maturation of B-cells, which then differentiate and secrete antibodies. Activation of both B-and T-cells is dependent on specific antigenic determinants. B-cells produce antibodies that can bind directly to the surface of the protein, whereas helper T-cells recognize peptides that are derived from the protein and are presented by HLA class II molecules.

**Figure 1:**
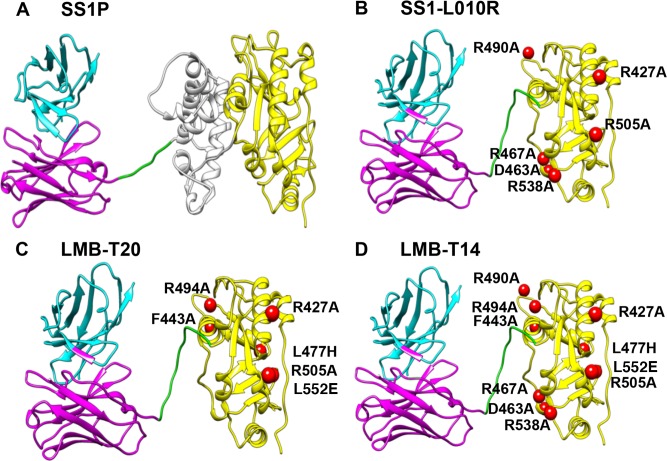
Structural models of RITs **A.** SS1P consists of the disulfide-stabilized heavy chain Fv (V_H_) (magenta) and light chain Fv (V_L_) (Cyan) of the antibody SS1P. The V_H_ is linked to a 38-kDa fragment of PE38 that is divided into domain II (gray), domain III (yellow), and part of domain Ib from native PE38. **B.** SS1-LO10R. 24-kDa fragment of PE24 with six point mutations in domain III designed to eliminate binding to B-cell receptor. Point mutations are marked with red balls. **C.** LMB-T20. PE24 with six point mutations in domain III designed to diminish T-cell epitopes. **D.** LMB-T14. PE24 with 10 point mutations in domain III designed to diminish B and T cell epitopes. All models are hypothetical arrangements based on the structures of native PE and immunoglobulin G; they do not represent actual structure determinations.

Mouse models have shown that elimination of murine B-cell epitopes can significantly reduce the formation of anti-drug antibodies (ADA) against therapeutic foreign proteins [9] and specifically against PE38 [10]. To identify the human B-cell epitopes in PE38, Liu et al. screened a phage display library that contained the Fv portions of antibodies isolated from B-cells of patients who had made anti-SS1P antibodies after treatment with SS1P. These Fvs were used to identify the human B-cell epitopes in domain III and mutations identified that suppressed these epitopes [11]. Finally this information was used to make a new mutant RIT (SS1-LO10-R),which has a deletion of domain II and six mutations in domain III (Figure 1). This immunotoxin has high cytotoxic activity and greatly reduced antigenicity, but it has a short serum half-life, because of its small size. To increase half-life and further decrease immunogenicity, the mouse Fv was replaced with a larger humanized anti-mesothelin Fab, resulting in an immunotoxin (RG7787) with a molecular weight of 72-kDa (Figure 1). RG7787 has recently entered clinical trials.

Elimination of T-cell epitopes is also a well-accepted strategy to de-immunize protein therapeutics. Yeung et al. showed that elimination of a T-cell epitopes in the protein IFNβ resulted in elimination of ADA response in BALB/c mice [12]. Similarly, we recently demonstrated that elimination of two murine T-cell epitopes in SS1P resulted in elimination of anti-SS1P antibodies in mice [13].

We previously reported the location of the eight human T-cell epitopes in the PE38 portion of immunotoxins [14] and used this information to construct LMB-T20, a RIT that targets mesothelin and has 80% of its T-cell epitopes diminished by introducing six point mutations in domain III and deleting a large portion of domain II [15]. The goal of this study was to make an immunotoxin reacting with mesothelin expressing cancer cells that has high cytotoxic and anti-tumor activity, and is optimized for minimal reactivity with the adaptive immune system by suppressing both B- and T-cell epitopes.

## RESULTS

### Design of de-immunized RITs targeting mesothelin

To construct the new de-immunized RIT(LMB-T14) we used the dsFv and toxin present in SS1P, deleted most of domain II and made mutations in domain III as shown Figure 1. SS1P (Figure 1A) is composed of an anti-mesothelin dsFv fused to a 38-kDa fragment of *Pseudomonas* exotoxin A (PE38). PE38 is made up of two domains; domain II (amino acids 253-364) contains a furin cleavage site necessary for toxin processing, and domain III (amino acids 395-613) contains the ADP ribosylation activity. Previous work showed that modifying SS1P by deletion of the majority of domain II and retaining the 11 amino acid furin cleavage site followed by a GGS spacer results in a RIT (SS1-LR-GGS) with high cytotoxic activity on many cell lines and decreased nonspecific toxicity in mice [16].

SS1-LO10R is derived from SS1-LR-GGS; it has six mutations in amino acids that suppress human B-cell epitopes (Figure 1B). Table 1 shows that SS1-LO10R has good cytotoxic activity with an IC_50_ of 1.7 pM, which is similar to that of the parent RIT that has no point mutations (SS1-LR-GGS) when evaluated on A431/H9 cells. LMB-T20 (Figure 1C) contains the same deletion in domain II as SS1-LO10R and six mutations in domain III that suppress T-cell epitopes [15]. LMB-T20 also has very good cytotoxic activity on A431/H9 cells (Table 1) with an IC_50_ of 2.2 pM.

#### Combination of B- and T-cell mutations

To make a cytotoxic protein with mutations in both B- and T-cell epitopes, we started with SS1-LO10R and introduced amino acid mutations that eliminate T-cell epitopes, usually one at a time as shown in Table 1. We previously observed that introduction of point mutation R494A in CD22 targeting RIT induces a 2-4-fold decrease in relative activity [14]. Here, similarly to the anti CD22 RIT, the mutation R494A (V3) resulted in a 4 fold decrease in activity (Table 1).

The activity of the intermediate construct was improved by the addition of F443A, which by itself induces a positive effect on the activity (V4), and when combined, it moderated the decrease in activity to 2.5-fold.LMB-T14 (Figure 1D) is the most de-immunized RIT. It includes a deletion of domain II and 10 point mutations in amino acids in domain III. Despite all the changes, it maintained very high cytotoxic activity, although a little less than LMB-T20 and LO10R.

**Table 1:** In vitro cytotoxic activity of various de-immunized RIT constructs in A431/H9 cells

RIT name	Total mutations	R427A	D463A	R467A	R490A	R505A	F443A	L477H	R494A	R538A	L552E	IC_50_ (pM)*	Relative activity to LMB-T20 (%)	Relative activity to LO10R (%)
		B+T	B	B	B	B+T	T	T	T	B	T			
LR-GGS	0											1.4	157	121
V1 (LO10R)	6	+	+	+	+	+				+		1.7	129	100
V2 (LMB-T20)	6	+				+	+	+	+		+	2.2	100	77
V3	7	+	+	+	+	+			+	+		7.2	31	24
V4	7	+	+	+	+	+	+			+		1.5	146	113
V5	8	+	+	+	+	+	+		+	+		4.3	51	40
V6	9	+	+	+	+	+	+	+	+	+		5.2	42	33
V7 (LMB-T14)	10	+	+	+	+	+	+	+	+	+	+	4.2	52	40
V8 (LMB-36)	8	+			+	+	+	+	+	+	+	4.5	49	38

* IC_50_ was evaluated in A431/H9 cells as described in the Experimental Procedures

### Cytotoxic activity on cells from mesothelioma patients

**Figure 2:**
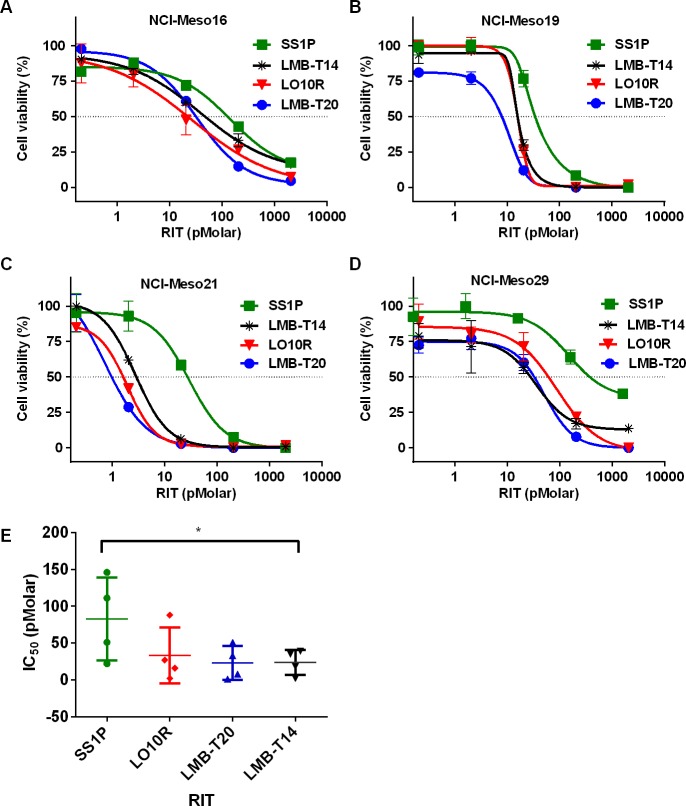
Activity of mesothelin targeting RITs on mesothelioma patients cells Cells cultured from the pleural fluid or ascites of four mesothelioma patients. NCI-Meso16 **A.**, NCI-Meso19 **B.**, NCI-Meso21 **C.**, and NCI-Meso29 **D.** were treated with increasing concentrations of RIT. After 72 hr, cells were evaluated for viability using a WST-8 assay and IC_50_ were calculated. **E.** Mean of the IC_50_ value for the four samples. Cells were treated in three replicas; line represents mean; error bar, SEM. Asterisk indicates significant differences of *p* < 0.05 (*).

Because SS1P has shown anti-tumor activity in patients with mesothelioma [6, 17], we established cell lines from mesothelioma patients and used them to examine the activity of the de-immunized variants. These cells resemble cells growing in patients more closely than established cell lines [18]. We found that LMB-T14 and its parent molecules (LMB-T20 and LO10R) were all more cytotoxic than SS1P with IC_50_s that were less than 100 pM on NCI-Meso16, NCI-Meso19, NCI-Meso21, and NCI-Meso29, (Figure 2A-2D). Figure 2E, which contains averaged data from four assays, shows that LMB-T14 had similar activity to LMB-T20 and LO10R and significantly better cytotoxic activity than SS1P (Figure 2E) (*p* < 0.05 in one way ANOVA in Dunn's multiple comparison test).

### Cytotoxic activity on a variety of cells lines

**Figure 3:**
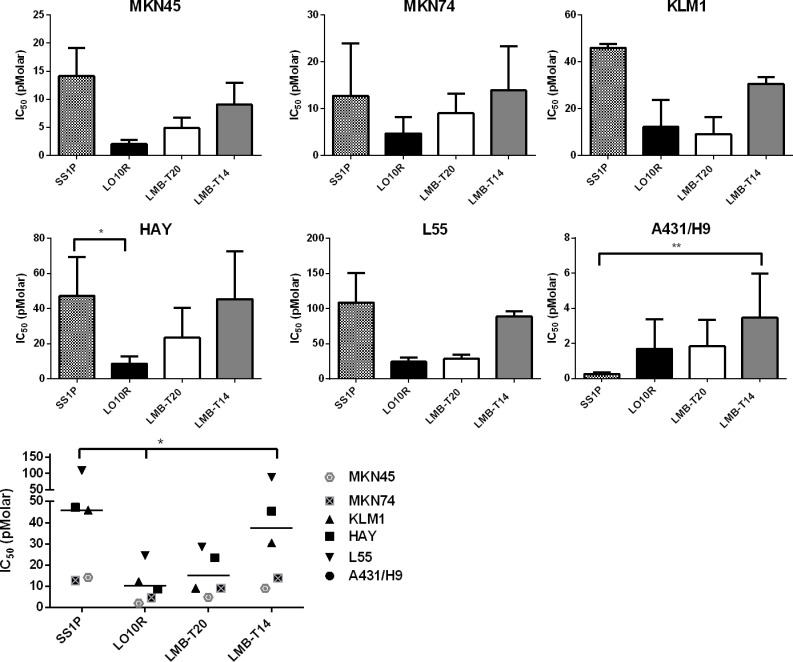
Cytotoxic activity in six mesothelin expressing cell lines The cytotoxicity of LMB-T14 was compared with SS1P, LMB-T20 and LO10R in a panel of six cells lines: HAY, L55, KLM1, MKN45, MKN74 and A431/H9. For each cell line, the mean IC_50_ of two or more assays is shown. Summary of all IC_50_s for all cell lines is shown in the bottom left. Error bars, SEM; *p* < 0.05 in the Freidman test with Dunn's multiple comparisons.

We also compared the activity of LMB-T14 with the three other RITs on several mesothelin expressing cancer cell lines (Figure 3). We found that all cell lines had good responses to LMB-T14; however, a loss in activity between LMB-T14 and its parent molecules (LO10R and LMB-T20) was observed in all cell lines. In stomach cell lines, MKN45 and MKN74, LMB-T14 had a small change in activity compared to LMB-T20 with 1.5-1.8-fold loss in activity. In HAY (mesothelioma cell line), L55 (lung cancer cell line) and KLM1 (pancreatic cell line) LMB-T14 had 3-5-fold lower activity than its parent molecules (LO10R and LMB-T20). Nevertheless, it had better activity than SS1P. Statistically significant differences are shown in Figure 3. In all the cell lines that naturally express mesothelin, SS1P was the least active (*p* < 0.01 in one way ANOVA with Dunn's multiple comparison). A431/H9 cells were the only cells that displayed a different pattern in which SS1P had a significantly better or similar activity to all of the variants. This is a transfected cell line that does not normally express mesothelin.

#### Functional stability

**Figure 4:**
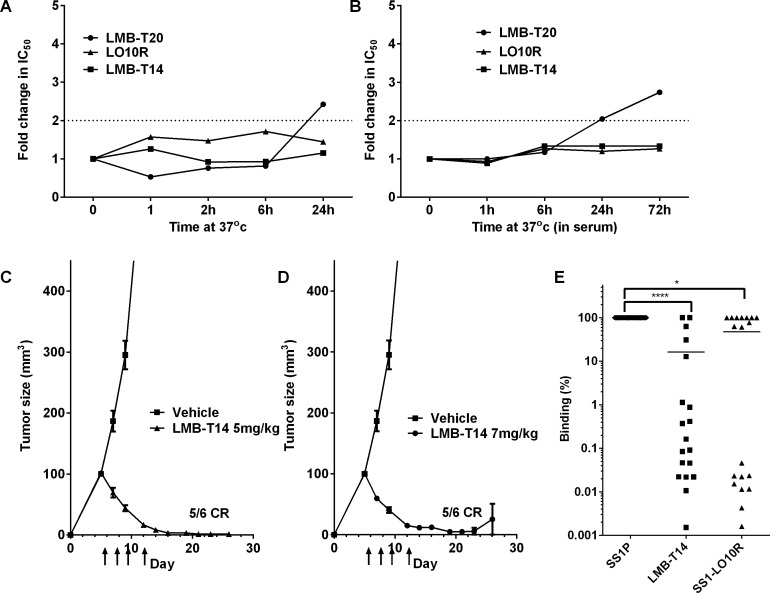
Stability, anti-tumor activity and antigenicity of LMB-T14 **A.**, **B.** Stability of RITs. RITs were warmed to 37°C for indicated durations and used to treat A431/H9 cells at serial concentrations. Cell viability was assayed, a curve fit was created for each RIT using a 4 parameter curve fit and IC_50_ was calculated. **A.** Fold change in IC_50_ after 0, 1, 2, 6 and 24 hr. **B.** Fold change in IC_50_ after incubation at 37°C in 100% human serum for 0, 1, 6, 24, and 72 hr. Cytotoxic activity was evaluated in six replicas for each data point with a standard deviation < 5% for all replicas. **C.**, **D.** Anti-tumor activity of LMB-T14 in mouse xenograft. A-thymic nude mice were innoculated 10^6^ A431/H9 cells at time 0. Intravenous treatment with LMB-T14 with a dose of 5 mg/kg **C.** or 7 mg/kg **D.** or vehicle began on day 5 and continued every other day for a total of four doses. On day 30 the experiment was terminated when 5/6 mice in both dose groups were tumor free. Arrows indicate the days that treatment was administered. **E.** Human antigenicity of SS1P and variant RITs. The reactivity of SS1P, LMB-T14 and LO10R with preexisting antibodies in human sera from 19 patients with neutralizing antibodies were compared using a binding assay to determine the concentration at which time the RITs reduced the signal of an ELISA to detect serum antibodies by 50% (IC_50_). The IC_50_ values of the RITs relative to SS1P are plotted. Line represents mean. Asterisks indicate significant differences of *p* < 0.0001 (****), *p* < 0.05 (*) in Friedman's test and Dunn's multiple comparisons.

To evaluate the stability of LMB-T14, we incubated LMB-T14 and the other RITs at 37°C for 1, 2, 6, and 24 or 72 hr in PBS at 0.5 mg/ml and evaluated their cytotoxic activity on A431/H9 cells. Cells were treated with various concentrations of each immunotoxin and an IC_50_was calculated for each time point. We found that LMB-T14 was very stable with no loss in activity after 24 hr (Figure 4A). LO10R also had excellent stability, whereas LMB-T20 was less stable and lost 2-fold activity in the time interval between 6 and 24 hr. To simulate the stability of LMB-T14 in the circulation of humans, we diluted it and the other variants in 100% human AB serum to 15μg/ml, incubated at 37°C for various times and measured cytotoxic activity. We found that LMB-T14 and LO10R were stable for 72 hr under these conditions and that LMB-T20 was less stable, losing 2-fold activity after 24hr and more after 72 hr (Figure 4B).

### Mouse toxicity

To evaluate the non-specific toxicity of LMB-T14, we treated small groups of Swiss mice with single doses of LMB-T14 and LMB-T20 (Table S1). We found that LMB-T14 was better tolerated than LMB-T20. There was no significant weight loss at a dose of 20 mg/kg, where as a similar dose of LMB-T20 was toxic in 4/4 mice.LMB-T14 was also well tolerated at a dose of 22 mg/kg, which is the highest tolerated dose reported for active immunotoxins in our lab. We found that 28 mg/kg was toxic for 4/4 mice. Interestingly, four QOD doses of 7 mg/kg (which adds up to 28 mg/kg) were well tolerated, with no weight loss. This indicates that 28 mg/kg is not toxic when administered over a period of time and suggests that LMB-T14 would be more efficacious if given in multiple small doses than in a single large dose.

### Efficacy of LMB-T14 in a mouse xenograft model

To evaluate the anti-tumor activity of LMB-T14, we implanted A431/H9 tumors into the flank of athymic nude mice. Mice were treated with LMB-T14 on days 5, 7, 9 and 12 after tumor implantation with doses of 5mg/kg or 7 mg/kg (Figure 4C and 4D). While the tumors treated with vehicle grew rapidly, reaching an average of 800 mm^3^ within 14 days, the treated groups had a significant decrease in tumor size as early as two days after the first dose. The tumors continued to decrease in size and by day 16, 5/6 tumors in both groups were undetectable. The complete tumor regressions persisted until day 30 when the experiment was terminated. In addition, this dose was well tolerated with no weight loss in the treated animals (Table S1).

### Antigenicity

Because LMB-T14 has mutations that are designed to diminish binding to B-cell receptors and to antibodies in human serum, we compared the reactivity of LMB-T14 and SS1-LO10R with SS1P using serum from 19 patients, who had developed neutralizing antibodies after treatment with SS1P. Figure 4D shows that LMB-T14 had significantly reduced binding to human anti-sera, and the magnitude of the decrease ranged from very little to more than a 3-log decrease with a mean of 16% (*p* < 0.001 in Friedman's test and Dunn's multiple comparisons).LO10R also had significantly reduced binding compared to SS1P (*p* < 0.05 in Friedman's test and Dunn's multiple comparisons) (Figure 4D). These findings indicate that the B-cell epitopes in SS1P that bind to patients sera are significantly diminished in these proteins. No significant difference between LO10R and LMB-T14 was observed, which indicates that the addition of four T-cell mutations did not significantly affect the structure of the molecule.

### T-cell activation

**Figure 5:**
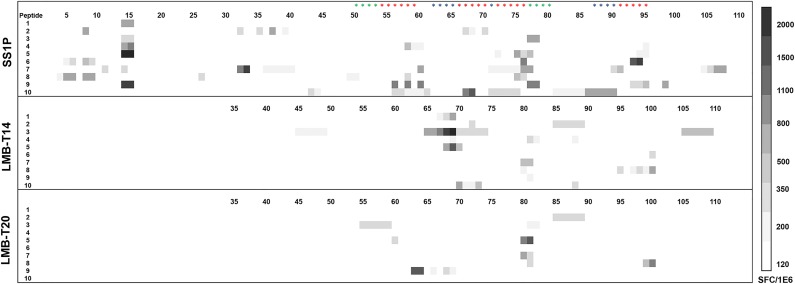
Stimulation of PBMC from 10 donors with LMB-T20, LMB-T14 and SS1P PBMC from 10 naïve donors were stimulated with either SS1P LMB-T14 or LMB-T20. After 14 days of *in vitro* expansion cells were re-stimulated with either 111 peptides spanning the sequence of PE38, 76 peptides spanning the sequence of LMB-T14 or LMB-T20, respectively. T-cell activation was detected using IL-2 ELISpot. Response strength is shown in the Spot Forming Cells ladder. Red stars represent mutations of T-cell epitopes, blue stars for B-cell mutations and green stars for both B and T-cell mutations.

To investigate the magnitude of the decrease in T-cell immunogenicity and to determine whether the four additional mutations (designed to remove B-cell epitopes) induced formation of new T-cell epitopes, we stimulated peripheral blood mononuclear cells (PBMC) from 10 normal donors with SS1P or the variants LMB-T20 and LMB-T14. After 14 days of *in vitro* expansion, the cells that were stimulated with SS1P were re-stimulated with 111 peptides spanning the sequence of PE38 and the cells that were stimulated with LMB-T14 or LMB-T20 were re-stimulated with 76 peptides spanning the sequence of LMB-T14 or LMB-T20, respectively. T-cell activation was detected using IL-2 ELISpot. As expected, both de-immunized RITs (LMB-T20 and LMB-T14) had a significant reduction in the number of IL-2 specific spots. The decrease was 61% with LMB-T14 and 81% with LMB-T20 (*p* < 0.01 in Wilcoxon matched-pairs signed rank test) (Figure 5).

Unexpectedly, 4/10 donors had a new and significant response to peptides 63-65 in LMB-T14 (Figure 5). Peptides 63-65 from LMB-T14 differ from WT and LMB-T20 by two separate mutations, which are D463A and R467A (labeled with blue stars). These mutations were introduced to eliminate B-cell epitopes. We further characterized this new epitope in 19 PBMC donors (Table S2) and searched for a correlation for specific HLA alleles. We found that 7/19 donors had a response to this epitope. The donors that responded to this epitope share three HLA DRB1 allele families: 15, 08, and 07.

## DISCUSSION

We describe here the properties of a new RIT, LMB-T14, that has greatly reduced immunogenicity because it contains mutations that suppress both T and B-cell epitopes. The new protein, which contains a large deletion of domain II and 10 amino acid mutations in domain III, is very cytotoxic to mesothelioma cells as well as other cancer cell lines, is well tolerated by mice and produces complete remissions of mesothelin expressing cancers in mice.

We have previously described immunotoxins with mutations in either B- or T-cell epitopes designed to decrease immunogenicity [11, 14]. In principle, elimination of either B- or T-cell epitopes should prevent immunogenicity. Unfortunately, total elimination of all B- or all T-cell epitopes is difficult to accomplish due to the complexity of the humoral immune system. One major obstacle is the polymorphism of HLA class II which makes it difficult to find single point mutations that will completely prevent the binding of the peptide-epitopes to various binding cores on the HLA. In addition, some of the mutations were able to decrease but not completely eliminate T-cell responses (Figure 5). Furthermore, the six mutations that eliminated B-cell epitopes do not decrease the binding of all tested human anti-sera [11]. In an attempt to improve on the properties of immunotoxins with only one arm of the immune system impaired, we have explored the possibility of incorporating mutations that decrease both B- and T-cell epitopes into one molecule. To our knowledge, this is the first time a therapeutic protein has been designed with silencing of both the B- and the T-cell epitopes.

Clinical trials with immune suppressive regimens also indicate a need to address both the B- and T-cells arms of the adoptive immune system. Hassan et al. treated patients with Rituximab to induce depletion of all circulating B-cells and followed with RIT treatment [19]. They found that B-cell depletion with Rituximab was not sufficient to prevent ADA formation against PE38. On the other hand, a combination of pentostatin and cyclophosphamide [6] that depleted both B- and T-cells prior to RIT treatment significantly delayed the ADA response, and allowed additional treatment cycles to be given. This finding indicates that both B- and T-cells are involved in the ADA response against RIT and that de-immunization against both should be beneficial.

In the process of combining six mutations designed to eliminate B-cell epitopes and the six mutations designed to diminish T-cell epitopes, we found that two point mutations (R427A and R505A) diminished both B- and T-cell mediated immunity. R505A and R427A have very large ASAs (150Å and 142Å) indicating that the arginines are located on the surface of the protein. Since B-cell epitopes are known to contain bulky hydrophilic amino acid like arginine [10, 20, 21], it is not surprising that R505A and R427A, which we found to suppress T-cell epitopes, also diminished B-cell epitopes. Others have previously reported that important immunogenic epitopes can be recognized by both B- and T-cells [22–24].

Our finding that the mutations (D463A and R467A) created a new T-cell epitope was unexpected, because alanine substitutions are frequently found to reduce the binding of a peptide to an HLA molecule due to loss of non-polar side chains [25, 26] and not to induce binding. The amino acid sequence that forms the new epitope is ARSQDLAAIWAGFYIAGD (peptides 64-65). A blast search of the mutant sequence (ARSQDLDAIWRGFYIAGD) revealed that the mutant did not resemble peptides found in other proteins except for the wild-type (WT) sequence. Similarly, a search in the immune epitope database (search for known epitopes with similar structure) [27] did not reveal similarity to other known epitopes. To eliminate this new epitope we constructed V8, which reverts residues 463 and 467 back to WT. LMB-36 has similar cytotoxic activity to LMB-T14 (Table 1). However reverting D463A and R467A back to WT also restores the B-cell epitope that those mutations eliminated. At this point it is not possible to determine which epitope (B or T) is more important for de-immunization. We also plan to determine if only one of the mutated amino acids is required for creating the new T-cell epitope.

When incorporating multiple point mutations into a molecule, there is the risk in decreasing its activity. There is a delicate balance between the decrease in activity the molecule will endure and the benefit of the de-immunization. This tradeoff is demonstrated in Table 1 that shows that addition of some of the de-immunizing mutations reduced the cytotoxic activity on several cancer cell lines, but not on cells from patients with mesothelioma (Figure 2). It is possible that LMB-T14 may be more efficacious in patients, due to its good stability, low nonspecific toxicity in animals so higher doses can be given and low immunogenicity will allow it to be given for more cycles.

## MATERIALS AND METHODS

### Human donor and patient samples

PBMC were isolated from apheresis samples from patients who were previously treated with a PE38-containing RIT and from naïve donors were collected under research protocols approved by the NIH Review Board (08-C-0026) and (99-CC-0168), respectively with informed consent. PBMC were isolated using gradient density separation by Ficoll-Hypaque (GE Healthcare, Piscataway, NJ) according to manufacturer's instructions. PBMC were frozen in 10% human AB serum (Gemini, Sacramento, CA) RPMI media (Lonza, Walkersville, MD) containing 7.5% DMSO (Cellgro, Manassas, VA) for 12 months in liquid nitrogen. Human sera were obtained under protocols 01-C-0011, 03-C-0243, and 08-C-0026.

### Peptide synthesis

Peptides for T-cell assays were synthesized by American Peptides (Sunnyvale, CA). All peptides were purified to 95% homogeneity by HPLC and confirmed by mass spectrometry.

### Construction, expression and purification of RIT

All RIT described in this work are composed of a heavy-chain Fv fused to LR-PE24 (VH-PE24) disulfide-linked to the light-chain Fv (VL) of SS1 antibody [28]. The different point mutations described in the constructs were added one by one using PCR overlap extension. The resulting PCR products were cloned back into the parent plasmid, and the mutations were confirmed by DNA sequencing. All RITs were purified by a standard protocol [29].

### Antigenicity assay

Binding of RITs to antibodies present in patients sera was assayed as previously described [10]. Briefly, ELISA plates were coated with 100 ng Fc-Mesothelin in 50 μl PBS over night at 4°C. In separate plates, the different RITs were incubated overnight at 4°C with patient's serum in serial concentrations. After washing of the coated plates, the immune complexes were transferred to the ELISA plates and incubated at room temperature for 1 hr. The human antibodies not bound to the RITs were captured by SS1P and detected. Next HRP-conjugated rabbit anti-human IgG Fc (Jackson Laboratory) was added, followed by TMB substrate (Thermo Scientific). IC_50_ values were calculated from the binding curves. The IC_50_ values indicate the concentration of RIT that inhibits 50% of the antibody reactivity with SS1P. The binding ratio was calculated from each IC_50_ value.

### *In vitro* expansion of PE38-specific cells and ELISpot assay

*In vitro* expansion using whole RIT and T-cell activation detection using IL-2 ELISpot were performed as previously described [30]. Briefly, PBMC from naïve donors were stimulated with 5μg/ml of SS1P, LMB-T14 or LMB-T20 in separate plates. The cells were supplemented with recombinant human IL-2 every 4 days (Millipore). On day 14, the cells were harvested and washed. They were brought to a concentration of 2×10^6^cells/ml and 50 μl were plated in pre-coated ELISpot plates (Mabtech). The enriched cells were then restimulated with peptide pools; cells that were expanded with SS1P were restimulated with 22 peptide pools spanning the sequence of WT PE38. Cells that were expanded using LMB-T14 or T20 were restimulated with 15 peptide pools spanning the sequence of the deimmunized RIT. Peptide pools that had a positive responses as defined [31] were fine screened to identify the individual immunogenic peptides by testing individual peptides from the pool.

### Cytotoxicity assay

#### Cytotoxicity of RIT against early passage mesothelioma tumor cells and cell lines

Early passage mesothelioma cells from the ascites or pleural fluid of four patients with mesothelioma seen at the National Cancer Institute on Institutional Review Board-approved protocols (08-C-0026) [18]. Frozen tumor cells were thawed, washed and grown in T75 flasks for 4 days in cell culture media. After reaching confluence (5×10^3^cells/well) cells were seeded in a 96 well plate and 24 hr later were treated with various concentrations of the RITs.

### Cytotoxic activity in established mesothelin expressing cell lines

Cells were seeded in a 96 well plate at optimal cell concentrations (A431/H9 cells 2.5 ×10^3^/well, KLM1, L55, MKN74, MKN45, and HAY cells at 5 ×10^3^/well) and 24hr later were treated with various concentrations of the RITs. The A431/H9 cell line was transfected in our laboratory and previously described [32]. The KLM1 pancreatic cell line was provided by Dr. U. Rudloff (NCI, Bethesda, MD), the L55 lung adenocarcinoma cell line was provided by Dr. S. Albelda (University of Pennsylvania, PA), MKN74 and MKN45 stomach cell lines were provided by Dr. T. Yamori (Pharmaceuticals and Medical Device Agency, Japan), and the HAY cells was provided by the Stehlin Foundation for Cancer Research (Houston, TX).

Cell viability was determined 72 hr later using WST8 cell counting kit (Dojindo Molecular Technologies Inc,) according to manufacturer's instructions. Color change was evaluated at O.D. 450nm.

Cell viability was normalized between 0-100 percent. Complete cell death (0%) was obtained by treating the cells with Cyclohexamideor Staurosporine and 100% by no treatment.

#### Functional stability assays

LMB-T20, LO10R and LMB-T14 were diluted in D-PBS to concentrations of 0.5 mg/ml. RIT variants were distributed in five aliquots and placed in an incubator at 37°C. Vials were taken out of the incubator and placed on dry ice for 15 min and transferred to −80°C at the following time points: 0, 1, 2, 6 and 24 hr. A431/H9 cells were plated in a concentration of 2.5 ×10^3^ cells/well and 24 hr later were treated using serial dilutions of the treated RITs in six replicas. Cell viability was detected 72 hr later as described above.

#### Serum stability

RIT variants were diluted to a concentration of 15 μg/ml in 100% human AB Serum (Gemini Bio-products). Five aliquots of 60 μl each were made for each protein and placed in 37°C.Vials were taken out of the incubator and placed in −20°C in the following time points: 0, 1, 6, 24 and 72 hr. Functional activity was evaluated as described above.

### Mouse xenograft tumor model

All animal experiments were performed in accordance with NIH guidelines and approved by the NCI Animal Care and Use Committee. Female athymic nude mice were injected subcutaneously in the flank with 1.0 × 10^6^ A431/H9 cells in 0.2 mL RPMI with 4 mg/mL Matrigel (BD Biosciences) on day 0. After 7 days, when the tumors reached 100 mm^3^, mice were injected IV with RITs in the indicated concentrations and the indicated schedules. Body weight and tumor size were observed for 30 days. Mice were euthanized if they experienced rapid weight loss or tumor burden greater than 10% body weight. No animals were excluded from statistical analysis. Tumor-size evaluation was evaluated blindly using a caliper.

### Nonspecific toxicity

Nonspecific toxicity was evaluated by IV injections of indicated doses to Swiss mice.

### Statistical analysis

Statistical analysis and plots were done using Graph Pad Prism software. For comparisons between two parametric variables we used Student T test. For comparisons between two non- parametric variables we used Wilcoxon matched rank test. For comparisons of multiple parametric variables we used One way ANOVA followed by Holm-Sidak's multiple comparisons test. For comparisons of multiple non-parametric variables we used Friedman test followed by Dunn's multiple comparison test. For comparisons between different cell lines and different RIT we used two way ANOVA with multiple comparisons with Dunnet test.

## SUPPLEMENTARY MATERIAL TABLES


