# Supplementary material for: Dual B- and T-cell de-immunization of recombinant immunotoxin targeting mesothelin with high cytotoxic activity
Source: Oncotarget. 2016 May 4;7(21):29916–26. doi: 10.18632/oncotarget.9171 (PMC5058652; doi:10.18632/oncotarget.9171)
Supplement: Supplementary file 1 [file oncotarget-07-29916-s001.pdf]

Dual B- and T-cell de-immunization of recombinant immunotoxin targeting mesothelin with high cytotoxic activity

Supplementary Material

Table S1. Tolerated and Non-Tolerated Doses of Variant RITs

	Dose (mg/Kg)	Frequency	Response	% Change in body weight	Number of mice
**LMB-T20**	18	1	Healthy	-1	4
	20	1	4/4 dead	-3	4
**LMB-T14**	20	1	Healthy	-3	4
	22	1	Healthy	-1	4
	28	1	4/4 dead	-1	4
	7	QOD x 4	Healthy	+1	6

Table S2. Response to Neu Epitope in Peptides 64-66

Donor	HLA	SS1P			LMB-T14			LMB-T20		
		64	65	66	64	65	66	64	65	66
51811	0804/1501	-	-	-	+++	+++	+++	-	-	-
21610	1501/1502	-	-	-	+++	+++	+	-	-	-
102609	0404/0802	-	-	-	+++	+++	++	-	-	-
71409	0301/0701/0716	-	-	-	-	-	+++	-	-	-
82009	1001/1501	-	-	-	+	++	-	-	-	-
80409	0701/1302	-	-	-	-	+	-	-	-	-
40610	0302/1503	-	-	-	-	+	-	-	-	-
60111	0301/0701	-	-	-	-	-	-	-	-	-
30911	0901/1301	-	-	-	-	-	-	-	-	-
22311	1201/1302	-	-	-	-	-	-	-	-	-
40110	0804/1302	-	-	-	-	-	-	-	-	-
40611	1201/1602	-	-	-	-	-	-	-	-	-
62211	0101/0301	-	-	-	-	-	-	-	-	-
30910	0301/0401	-	-	-	-	-	-	-	-	-
120809	1101/1301	-	-	-	-	-	-	-	-	-
60811	0101/1301	-	-	-	-	-	-	-	-	-
12610	0401/0404	-	-	-	-	-	-	-	-	-
012913A	0301/0401	-	-	-	-	-	-	-	-	-
121009	0101/11	-	-	-	-	-	-	-	-	-

- <100 SFC/1E6 cells
+ 100 300 SFC/1E6 cells
++ 300 600 SFC/1E6 cells
+++ >600 SFC/1E6 cells
